# Drinks containing anthocyanin-rich blackcurrant extract decrease postprandial blood glucose, insulin and incretin concentrations^☆^^☆☆^^★^

**DOI:** 10.1016/j.jnutbio.2016.09.002

**Published:** 2016-12

**Authors:** Monica L. Castro-Acosta, Leanne Smith, Rosalind J. Miller, Danielle I. McCarthy, Jonathan A. Farrimond, Wendy L. Hall

**Affiliations:** a Diabetes & Nutritional Sciences Division, King's College London, Franklin-Wilkins Building, 150 Stamford Street, London, SE1 9NH, UK; b GlaxoSmithKline Services Unlimited, GSK House, 980 Great West Road, Middlesex, TW8 9GS, UK; c Suntory Beverage and Food Europe Ltd, 2 Longwalk Road, Stockley Park, Uxbridge UB11 1BA, UK

**Keywords:** AOB, Area over baseline, Cmax, Maximum concentration, CON, Control treatment, DVP-SI, Digital volume pulse stiffness index, DVP-RI, Digital volume pulse reflection index, GIP, Glucose-dependent insulinotropic polypeptide, GLP-1, Glucagon-like peptide-1, H-BE, High dose of blackcurrant extract, L-BE, Low dose of blackcurrant extract, M-BE, Medium dose off blackcurrant extract, NEFA, Non-esterified fatty acids, TAG, Triacylglycerol, Tmax, Time of maximum concentration, Randomized controlled trial, Postprandial glycemia, Incretins, Insulin, Anthocyanins, Polyphenols, Berries

## Abstract

Blackcurrants are rich in polyphenolic glycosides called *anthocyanins*, which may inhibit postprandial glycemia. The aim was to determine the dose-dependent effects of blackcurrant extract on postprandial glycemia. Men and postmenopausal women (14 M, 9 W, mean age 46 years, S.D.=14) were enrolled into a randomized, double-blind, crossover trial. Low sugar fruit drinks containing blackcurrant extract providing 150-mg (L-BE), 300-mg (M-BE) and 600-mg (H-BE) total anthocyanins or no blackcurrant extract (CON) were administered immediately before a high-carbohydrate meal. Plasma glucose, insulin and incretins (GIP and GLP-1) were measured 0–120 min, and plasma 8-isoprostane F_2α_, together with arterial stiffness by digital volume pulse (DVP) was measured at 0 and 120 min. Early plasma glucose response was significantly reduced following H-BE (*n*=22), relative to CON, with a mean difference (95% CI) in area over baseline (AOB) 0-30 min of −0.34 mmol/l.h (−0.56, −0.11, *P*<.005); there were no differences between the intermediate doses and placebo. Plasma insulin concentrations (AOB 0–30 min) were similarly reduced. Plasma GIP concentrations (AOB 0–120 min) were significantly reduced following H-BE, with a mean difference of −46.6 ng/l.h (−66.7, −26.5, *P*<.0001) compared to CON. Plasma GLP-1 concentrations were reduced following H-BE at 90 min. There were no effects on 8-isoprostane F_2α_ or vascular function. Consumption of blackcurrant extract in amounts roughly equivalent to 100-g blackcurrants reduced postprandial glycemia, insulinemia and incretin secretion, which suggests that inclusion of blackcurrant polyphenols in foods may provide cardio-metabolic health benefits. This trial was registered at clinicaltrials.gov as NCT01706653.

## 1 Introduction

Evidence is emerging that fruit polyphenols may have wide-ranging beneficial effects on vascular and metabolic health [1]. Among the long list of potential fruit bioactives are the anthocyanins: polyphenolic glycosides belonging to the flavonoid family that vividly pigment berries and other plant foods. The aglycones of anthocyanins, anthocyanidins, include delphinidin, petunidin, cyanidin, malvidin, pelargonidin and peonidin. Glycosylation can involve glucose, galactose, rhamnose and arabinose or various combinations of these sugars. There may also be acylation with *p*-coumaric, ferulic, caffeic, sinapic, malonic, acetic or *p*-hydroxybenzoic acids. Blackcurrants are particularly rich in delphinidin-3-rutinoside, cyanidin-3-rutinoside, delphinidin-3-glucoside and cyanidin-3-glucoside, as well as condensed tannins (proanthocyanidins), oligomeric and polymeric chains of flavan-3-ols [2].

Since dietary anthocyanins are derived from a relatively narrow range of specific foods, average intakes are difficult to establish from short-term food records. Mean estimates from food frequency questionnaires are 12.5 to 15.2 mg/d (IQR: 4.6–19.3 mg/d) in US middle-aged and older men and women, mainly from strawberry and blueberry consumption [3], and 17.7 mg/d (IQR: 8.4–23.6 mg/d) in UK women, mainly from grapes, pears, wine, strawberries and raspberries [4]. Epidemiological studies have reported that higher intakes of anthocyanins are associated with a reduced risk of hypertension and arterial stiffness [3], [4] and lower markers of systemic inflammation and insulin resistance [5]. In support of a beneficial effect of habitual anthocyanin intake on insulin resistance, higher intakes of berries (4 d food records) at baseline were associated with a reduced risk of Type 2 diabetes at follow up (mean 19.3 years) in a Finnish prospective cohort of middle-aged and older men [6].

Ingestion of berry anthocyanins may have rapid effects on blood glucose levels by delaying carbohydrate digestion and inhibiting the rate of glucose absorption across the intestine [7], [8], [9], [10], [11], [12], [13], although proanthocyanidins are likely to be responsible for inhibition of α-amylase [13]. Randomized controlled trials suggest that consuming whole berries may help control blood glucose concentrations [14], [15], [16], [17], [18], but the active component is not yet clear.

The present study set out to test the hypothesis that drinks containing an anthocyanin-rich blackcurrant extract would decrease postprandial peak glucose concentrations following a carbohydrate meal in a dose-dependent fashion, compared with a matched placebo drink. Secondary outcomes included plasma insulin, GIP and GLP-1 response. Reducing postprandial glycemia may reduce markers of oxidative stress and endothelial function [19], [20]; therefore, changes in indices of vascular function and plasma 8-isoprostane F_2α_ concentrations were also evaluated.

## 2 Subjects and methods

### 2.1 Subjects

Ethical approval for the study was obtained from King's College London research ethics committee (reference BDM/11/12–88), and written informed consent was given by participants. The trial was carried out in accordance with the Declaration of Helsinki of 1975 as revised in 1983 and registered at ClinicalTrials.gov (NCT01706653). The aim was to recruit 22 subjects, allowing for four drop-outs to complete 18 subjects, which would enable detection of a 1.6-mmol/l difference in glucose incremental Cmax, assuming 80% power (estimated from Torronen *et al*. [17] treatment group S.D. of 1.3 to 1.5 mmol/l for Cmax, rho=0.1), using a paired *t* test with an alpha 0.017 (allowing for three-way comparisons). Social networking Web site advertisements and a circular email within King's College London were used for recruitment, which was initiated in August 2012. A participant information sheet was provided to volunteers who expressed interest. Participants, recruited from King's College London and from the general public in the London area, attended the Metabolic Research Unit at the Diabetes & Nutritional Sciences Division, King's College London, in a fasting state for a screening appointment which included the measurement of height, weight, waist circumference, % body fat (by bioelectrical impedance using the Tanita™ Body Composition Analyser), seated blood pressure, liver function tests, glucose, lipid profile and haematology. A small remuneration was given for participation in the study. Inclusion criteria were healthy men aged 20–60 years and postmenopausal women aged 45–60 years, BMI 18–35 kg/m^2^, able to understand the information sheet and willing to comply with study protocol and able to give written informed consent. Women aged 45 years or older who reported not having had a period for 12 months or longer were defined as postmenopausal. Exclusion criteria were as follows: phenylketonuria; allergy, hypersensitivity or intolerance to any foods/food ingredients; participation in another clinical trial; those with full blood counts and liver function tests outside of the normal range; current smokers or those who gave up smoking within the last 6 months; reported medical history of cardiovascular disease, cancer, liver, kidney or bowel disease; fasting glucose≥7.1 mmol/l or uncontrolled Type 2 diabetes; presence of gastrointestinal disorder or use of drug which is likely to alter gastrointestinal motility or nutrient absorption; history of substance abuse or alcoholism; unwilling to restrict consumption of specified high polyphenol foods for 24 h before the study; weight change of >3 kg in preceding 2 months; body mass index<18 and >35 kg/m^2^; fasting blood cholesterol≥7.5 mmol/l; fasting TAG≥5 mmol/l; blood pressure≥160/100 mmHg; current use of medications that may interfere with the study such as alpha-glucosidase inhibitors (*e.g.*, acarbose), insulin-sensitizing drugs (*e.g.*, metformin, thiazolidinediones), sulfonylureas, and lipid-lowering drugs; current use of nutritional supplements that may interfere with the study such as higher dose vitamins/minerals (>200% RNI), B vitamins, Vitamin C, calcium, copper, chromium, iodine, iron, magnesium, manganese, phosphorus, potassium and zinc.

### 2.2 Study design

A randomized, double-blind, crossover design was used to compare four test drinks, with study visits scheduled at least 1 week apart. Each test drink consisted of a “no added sugar” fruit drink with increasing doses of blackcurrant extract added to it, providing 90 kJ, <0.1-g protein, <5-g carbohydrate and 0.4-g fat. The blackcurrant extract (BerryPharma® by Iprona AG, Lana, Italy) contained 5775-mg total polyphenols per 100-ml extract (2822-mg total anthocyanins per 100 ml), analysed by RSSL The Lord Zuckerman Research Centre (Reading, UK); see Table 1. The drinks were formulated by Döhler (Milton Keynes, UK) and GlaxoSmithKline Nutritional Healthcare R&D (Coleford, UK), later to become Lucozade Ribena Suntory (Uxbridge, UK), and standardized to contain 600-mg anthocyanins (H-BE), 300-mg anthocyanins (M-BE) or 150-mg anthocyanins (L-BE). The placebo drink did not have any blackcurrant extract added to it (CON), but M-BE, L-BE and CON were adjusted with added tannins to ensure blinding by making sure each of the drinks had equivalent astringency/bitterness in taste. This meant that CON also contained tannin polyphenols. The standardized high carbohydrate meals consumed on each study visit consisted of 100-g thick sliced white bread (Hovis, London, UK) with 32-g Hartley's smooth apricot jam (Hain Daniels Group, Leeds, UK), both high carbohydrate foods which are low in phenolics (<6-mg/100-g fresh weight) [2], to provide 1127-kJ energy, 62-g carbohydrate, 39-g starch, 23-g sucrose. The total carbohydrate intake including the test drink was 67 g. The randomization schedule was generated by independent contracted parties within GlaxoSmithKline Nutritional Healthcare, and allocation to treatment sequence was carried out once a participant was determined to be eligible and had agreed to take part in the study according to the schedule of randomly generated treatment sequences. Allocation of treatment at each visit was blinded from the investigators, laboratory technicians, statisticians and the study participants.

**Fig. 1:**
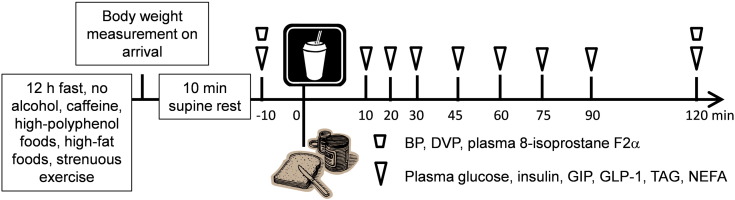
Study visit protocol.

The participants consumed each of the four test drinks delivered in opaque bottles and consumed with a straw within 2 min, in random order, after baseline measurements/blood samples were taken. The mixed carbohydrate meal was consumed immediately after consumption of the drink, within 5 min. Each test meal protocol (Fig. 1) was separated by at least 1 week.

On the day preceding each test meal, participants were told not to participate in strenuous exercise and to avoid alcohol, caffeine, oily fish, high polyphenol foods (from a list provided) and foods high in fat. They were provided with a standardized low-fat meal (containing <10 g fat) as their evening meal, which they were required to consume before 20 h and then to avoid eating or drinking anything except for water until the study visit (a 12-h fast). Participants attended the metabolic research unit between 08 h and 10 h 30 min the next day. They were asked to empty their bladder, be weighed and then rest in a supine position in a temperature-controlled, quiet, dim room for 10 min before blood pressure and digital volume pulse (DVP) measurements were taken. A cannula was inserted into the forearm antecubital vein, and blood was collected for baseline analysis (in duplicate at −15 and −10 min for glucose). Following consumption of the test meal, further venous blood samples were collected 10, 20, 30, 45, 60, 75, 90 and 120 min after the test drink/meal for plasma glucose, insulin, NEFA, GIP, GLP-1 and TAG analysis and at 120 min only for 8-isoprostane F_2α_ analysis. Blood pressure/vascular measurements were made within 10 min after the final blood sample had been taken at 120 min. Participants had access to water to sip as required over the 2-h period. Adverse events were recorded at all study visits where necessary by asking the following question during each visit, including any follow-up visits or telephone calls and documenting the reply: “Have you felt unwell, experienced any symptoms or taken any medication today or since the last session?”

### 2.3 Methods

Blood samples for plasma GIP and GLP-1 analysis were collected into EDTA tubes (Becton Dickinson, UK) and centrifuged at 1300 ×*g* for 15 min at 4 °C, and plasma was stored at −80 °C until analysis. EDTA tubes for GLP-1 analysis had 10 μl per ml blood dipeptidyl peptidase iv inhibitor added (Millipore, MO, USA). GIP and GLP-1 were determined by ELISA kits (Millipore Corporation, MA, USA). Further blood samples were collected into fluoride oxalate tubes for glucose analysis and SST™ II tubes for TAG, insulin and NEFA analysis; plasma and serum were stored frozen at −40 °C until analysis (Becton Dickinson, UK). Enzymatic assays were used to determine concentrations of NEFA, glucose and TAG (TAG and glucose: Instrumentation Laboratory, cat.no. 0,018,255,640 and cat.no. 00,018,250,740, Warrington, Cheshire, UK; NEFA C: Wako Chemicals GmbH, cat.no. 999–75,406, Neuss, Germany) on an ILAB-650 analyser (Instrumentation Laboratory, Warrington, UK). Blood for 8-isoprostane-F_2α_ analysis was drawn into chilled citrated tubes (Becton Dickinson, UK), and chilled fresh indomethacin (cyclooxygenase inhibitor) was immediately added (final concentration 15 μmol/l). The sample was kept on ice 30 min prior to centrifugation at 2400 ×*g* for 15 min. BHT was added (final concentration 20 μmol/l), and the samples were frozen in liquid N_2_ and stored at −80 °C until analysis of 8-isoprostane F_2α_ by GC/MS as previously described [21].

Blood pressure was measured according to British Hypertension Society guidelines using an automated upper arm blood pressure monitor, the Omron 705IT (Omron Healthcare Europe B.V.). DVP was obtained by photoplethysmography (PulseTrace, Micro Medical Ltd., Kent, UK) and used to calculate stiffness index (DVP-SI, m/s) and reflection index (DVP-RI, %).

### 2.4 Statistical analyses

Mean values for plasma glucose concentrations were calculated from duplicate measurements made at baseline (−15 and−10 min) before statistical analysis. A linear mixed effects model was used to analyse incremental Cmax and AOB using PROC MIXED in SAS software (Marlow, UK). Main effects of drink and drink × time interactions for the change from baseline at each time point were calculated by linear mixed effects modelling using SPSS Statistics Version 21 (IBM, UK). The models included subject as a factor (a random effect), fixed factors were drink (and time and drink × time interaction where appropriate) and period. Baseline values and two baseline terms were included as covariates: (a) subject-level baseline; the number of valid responses calculated as the mean baseline across all periods within a subject, and (b) the period-level baseline minus the subject-level baseline. *P*-values were adjusted using Dunnett's procedure for the comparison against a control (reference) product, either using SAS for incremental Cmax and AOB, or using two-way repeated measures ANOVA in GraphPad Prism Version 6.00 for Windows (GraphPad software, CA, USA) for drink and drink × time effects (since SPSS does not provide Dunnett's multiple testing adjustment in the linear mixed model facility with repeated measures). The assumption of normality and homogeneity of variance was investigated. Violation of these assumptions were overcome where appropriate using natural logarithmic transformation. Tmax data were analysed by Friedman's nonparametric test using GraphPad Prism.

## 3 Results

**Fig. 2:**
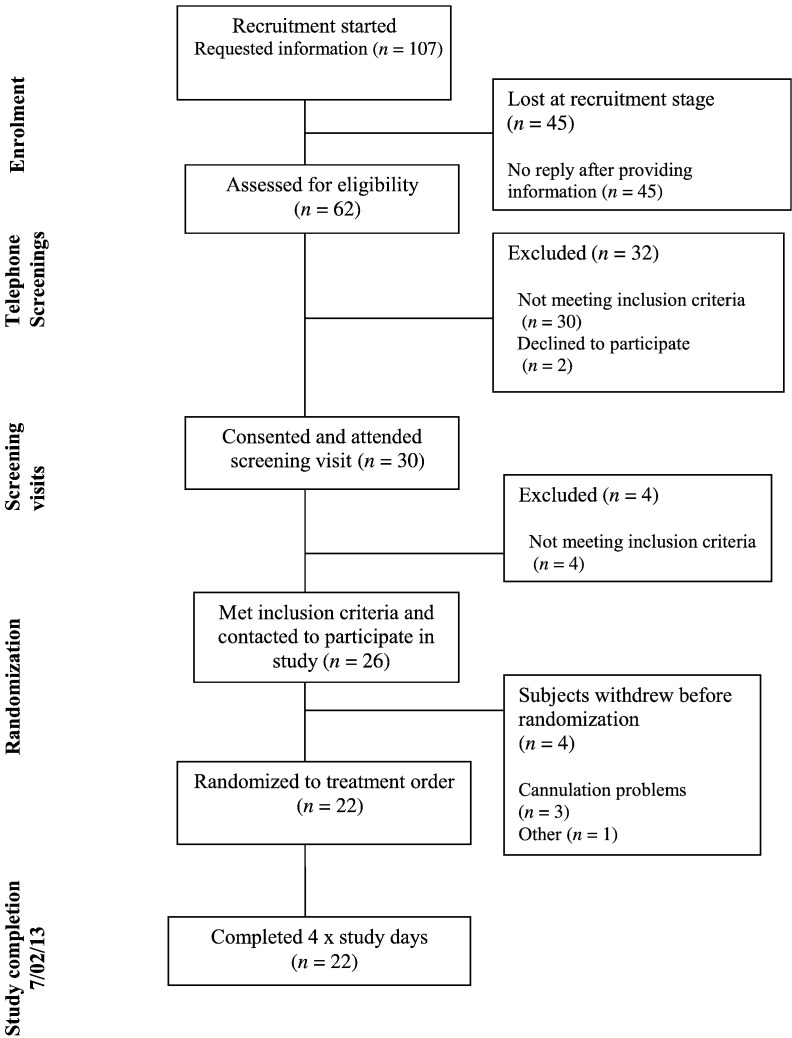
Consort diagram.

Thirty volunteers who met the initial eligibility criteria following a telephone questionnaire attended screening sessions at King's College London, of whom 4 did not meet inclusion criteria. Of the 26 eligible participants, 22 were randomized to treatment and completed the study. All completing subjects were fully compliant with the study protocol. Details are shown on the consort diagram (Fig. 2). The characteristics of those who completed the study are shown in Table 2. There were three treatment emergent adverse events: one was considered treatment-related (discoloured faeces following CON), and the others (following H-BE) were related to cannulation site discomfort. No adverse events were considered serious.

### 3.1 Glucose, insulin and incretins

**Fig. 3:**
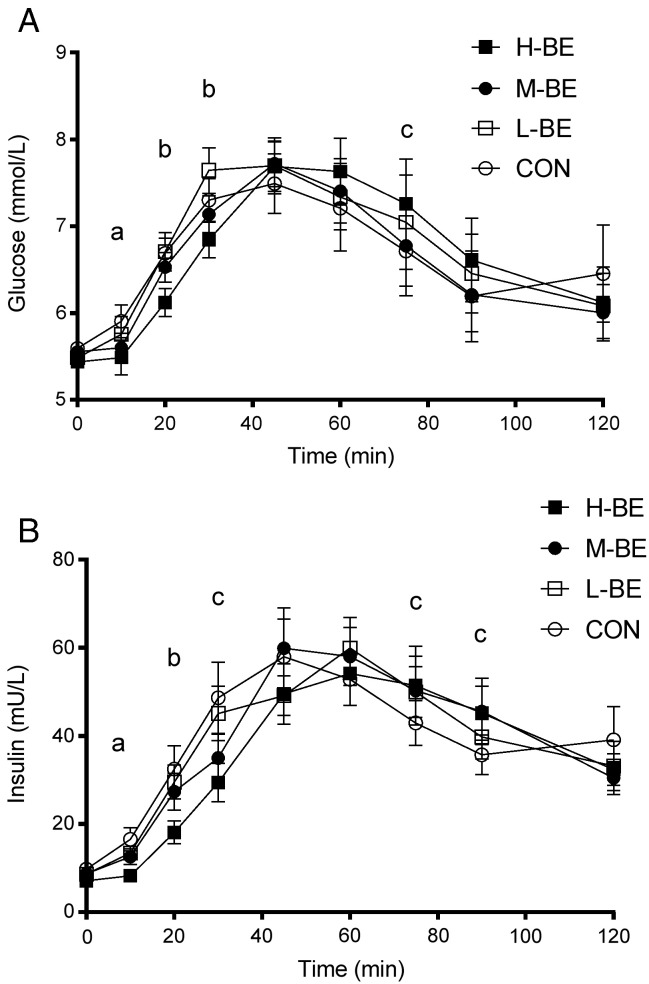
Postprandial plasma glucose and insulin concentrations. Mean (±S.E.M.) plasma glucose (A) and insulin (B) concentrations following ingestion of four low sugar fruit drinks 2 min before consuming a mixed carbohydrate meal, in randomized order: H-BE: high blackcurrant extract; M-BE: medium blackcurrant extract; L-BE: low blackcurrant extract; CON: placebo. *N*=22. All data were natural log transformed before mixed model analysis. For glucose *P*<.001 for an overall drink effect on changes from baseline, and for insulin, *P*<.001 for a drink effect and *P*<.001 for a drink x time interaction on raw values and changes from baseline. (A) *post hoc* analysis of timepoint differences in change from baseline in glucose compared to CON with Dunnett's adjustment: ^a^*P*<0.05 for the difference between H-BE and CON and M-BE and CON; ^b^*P*<.005 for the difference between H-BE and CON; ^c^*P*<0.01 for the difference between H-BE and CON. (B) *post hoc* analysis of timepoint differences in change from baseline in insulin with Dunnett's adjustment: ^a^*P*<.005 for the difference between H-BE and CON; ^b^*P*<0.01 for the difference between H-BE and CON; ^c^*P*<0.05 for the difference between H-BE and CON.

The postprandial changes in plasma glucose after the test drinks and standard carbohydrate meals are shown in Fig. 3A. An overall drink effect for the change from baseline of (*Ln*)glucose concentrations 0–120 min (*P*<.05) was observed, with no statistically significant drink × time interaction for postprandial glucose response (*P*=.16). As Fig. 3A illustrates, H-BE inhibited glucose concentrations during the initial 30 min of the postprandial period (AOB 0–30 min mean difference H-BE *vs*. CON (95% CI) −0.34 mmol/l.h (−0.56, −0.11, *P*<.005); AOB 0–120 min were not significantly different between meals. *Post hoc* pairwise comparisons showed that there were significantly lower glucose concentrations following H-BE compared to CON at 10–30 min postdrink (Fig. 3A), and there was a statistically significant increase in glucose following H-BE at 75 min relative to CON (mean difference in change from baseline values was 0.72 mmol/l (0.18, 1.25; *P*<.01). Cmax (adjusted for baseline) was not significantly different between meals. Tmax was slightly greater following H-BE (mean at 55 min, 95% CI 48, 62) compared to M-BE, L-BE and CON (means were 46 min, 39, 62; 49 min, 42, 57; 48 min, 36, 60, respectively), but this did not reach statistical significance (*P*=.06).

There was a statistically significant drink effect (*P*<.001) and drink × time interaction (*P*<.05) for (*Ln*)insulin 0–120 min, and a drink × time interaction (*P*<.05) for the change from baseline in (*Ln*)insulin 0–120 min. *Post hoc* analysis showed similar temporal drink differences to glucose (Fig. 3B), with significantly lower insulin concentrations initially, following H-BE compared to CON, at 10, 20 and 30 min, and higher concentrations at 75 and 90 min (Fig. 3B). The mean difference in AOB 0–30 min between H-BE and CON (95% CI) was −8.77 mU/l.h; −13.86, −3.68, *P*<.005. There were no differences in Cmax, Tmax and AOB 0–120 min for insulin.

**Fig. 4:**
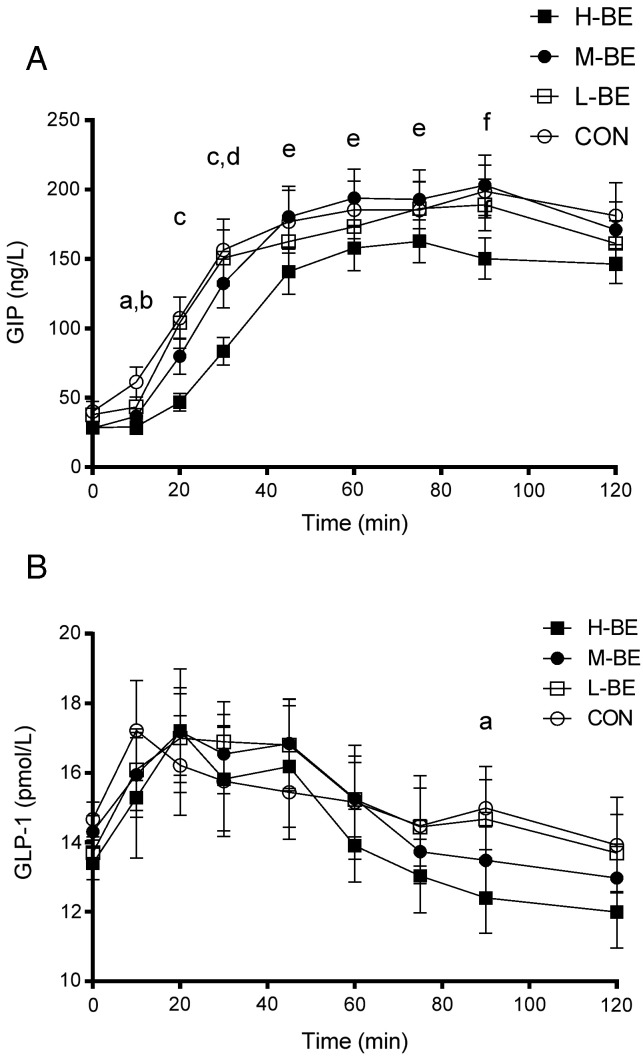
Postprandial plasma GIP and GLP-1 concentrations. Mean (±S.E.M.) plasma GIP (A) and GLP-1 (B) concentrations following ingestion of four low sugar fruit drinks 2 min before consuming a mixed carbohydrate meal, in randomized order: H-BE: high blackcurrant extract (GIP *n*=17; GLP-1 *n*=20); M-BE: medium blackcurrant extract (GIP *n*=20; GLP-1 *n*=22); L-BE: low blackcurrant extract (GIP *n*=20; GLP-1 *n*=21); CON: placebo (GIP *n*=19; GLP-1 *n*=22). All data were natural log transformed before mixed model analysis. For GIP, there was an overall drink effect on raw values (*P*<.0001) and changes from baseline (*P*<.0001) and a drink × time interaction on raw values (*P*<0.005), and for GLP-1, there was an overall drink effect on raw values and changes from baseline (*P*<.001). (A) *post hoc* analysis of timepoint differences in change from baseline in GIP with Dunnett's adjustment: ^a^*P*<0.0001 for the difference between H-BE and CON and ^b^*P*<0.001 for the difference between L-BE and CON; ^c^*P*<0.0005 for the difference between H-BE and CON; ^d^*P*<0.005 for the difference between H-BE and L-BE and H-BE and M-BE; ^e^*P*<0.05 for the difference between H-BE and CON; and ^f^*P*<0.0005 for the difference between H-BE and CON. (B) *post hoc* analysis of timepoint differences in change from baseline in GLP-1 with Dunnett's adjustment: ^a^*P*<0.05 for the difference between H-BE and CON.

Plasma GIP changes from baseline concentration were significantly reduced during the whole postprandial measurement period following H-BE anthocyanins compared to CON, with a significant decrease in Cmax (mean difference, −64.4 ng/l; −95.5, −33.4; *P*<.0005) and AOB 0–120 min (*P*<.0001) and highly statistically significant drink effect (*P*<.0001) and drink × time interaction (*P*<.005) on (*Ln*)GIP 0–120 min and change from baseline of (*Ln*)GIP 0–120 min (drink effect *P*<.0001). *Post* *hoc* pairwise comparisons with Dunnett's adjustment for multiple comparisons showed consistently reduced plasma GIP concentrations at all timepoints following H-BE up until 90 min (Fig. 4A).

Plasma GLP-1 concentrations were also reduced by H-BE (Fig. 4B), with a statistically significant drink effect on changes from baseline of (Ln)GLP-1 (*P*<.0005) but no significant drink × time interaction. *Post hoc* pairwise comparisons showed reduced plasma GLP-1 concentrations relative to baseline following H-BE compared to CON and L-BE anthocyanins at 90 min (Fig. 4B). There were no differences in Cmax, Tmax and AOB 0–120 min for GLP-1.

### 3.2 Lipids

Plasma TAG concentrations [(Ln)TAG up to 120 min] were significantly different according to drink, but this was due to a significant difference at baseline (*P*<.005), and the changes from baseline (Ln)TAG did not differ (Supplementary Fig. 1A). There was no effect of drink on plasma NEFA concentrations (Supplementary Fig. 1B).

### 3.3 Isoprostanes and vascular function

(*Ln*)8-isoprostane F_2α_ concentrations, blood pressure, DVP-SI and DVP-RI changes from baseline were not significantly different between drinks (Table 3).

## 4 Discussion

This study aimed to investigate whether blackcurrant anthocyanin-rich fruit drinks could inhibit the rise in blood glucose concentrations following a high-carbohydrate meal in a healthy population. The highest dose of blackcurrant anthocyanins inhibited the rate of the increase in plasma glucose, and insulin concentrations in the first 30 min had an inhibitory effect on plasma GIP concentrations up to 90 min postmeal and reduced plasma GLP-1 concentrations at ~90 min, but the lower doses had no significant inhibitory effects. This delaying effect on postprandial glycemia may, if experienced habitually over many years, reduce risk of developing insulin resistance and, eventually, Type 2 diabetes [22]. H-BE was equivalent to approximately 100-g fresh raw blackcurrants [2], which could conceivably be consumed without discomfort at one sitting. Furthermore, there tended to be a rebound effect on plasma glucose and insulin concentrations in the later timepoints, with the highest dose being associated with higher concentrations at 75–90 min, as observed previously with whole berry purée and nectar [15], [16]. This did not translate to the same pattern in the incretin levels, which consistently showed reduced concentrations following the highest dose compared to placebo throughout the 120-min postprandial period. No changes in secondary outcome variables related to lipids, vascular function or oxidative stress were observed.

These data are in agreement with previous reports that whole berry meals or berry nectars (juices) rich in anthocyanins, including those derived from blackcurrants, inhibited plasma glucose and insulin concentrations in the first 30 min following consumption [14], [15], [16], [17], [23]. Furthermore, these results provide novel data showing that a liquid blackcurrant extract (devoid of fibre and low in other confounding nutrients) not only lowers glucose and insulin concentrations but also inhibits incretin secretion. The observation that GLP-1 secretion may be inhibited in the latter half of the postprandial period is in contrast to a previous study of a mixed berry meal consumed with sucrose which showed that plasma GLP-1 concentrations were slightly increased in the first hour following consumption of the meal [16], which might reflect differences in study design such as the solid state of the carbohydrate load and mixed sucrose and starch composition of the present test meal.

The difference in carbohydrate type and physical state of the test meal might also explain why a previous study observed a return to baseline of insulin concentrations following their reference meal (sucrose and water) [15], whereas plasma insulin concentrations remained elevated following our control and blackcurrant extract test meals (drink, followed by bread and apricot jam). Consequently, the insulin-mediated suppression of circulating NEFA concentrations was not different in the current study, in contrast to the previous study which observed an inhibition of the upwards return to baseline in plasma NEFA concentrations around 90–120 min following blackcurrant puree and blackcurrant juice, in line with higher insulin concentrations at these times points in comparison to the reference meal [15].

Inhibition of intestinal glucose transporters (*e.g.*, SGLT1, GLUT2) and inhibition of digestive enzymes are the primary proposed mechanisms by which blackcurrant polyphenols might act on glycaemic response [10], [24], [25], although delayed gastric emptying cannot be ruled out. Polyphenolic components of blackcurrant extract such as proanthocyanidins may reduce pancreatic α-amylase activity in the duodenum by binding to the protein [13]. Additional inhibition of carbohydrate digestion may occur by interaction of anthocyanins and other blackcurrant polyphenols with intestinal brush border α-glucosidases, resulting in reduced maltase and sucrase activities [8]. Since our control drink and lower dose intervention drinks also contained added tannins in order to match the bitter taste of H-BE, there is likely to have been inhibition of α-amylase across all test drinks. Thus, differences observed here are possibly related to anthocyanin content. Anthocyanins can also be transported into the intestinal cell using the SGLT1 transporter, and competition for this route following an anthocyanin-rich meal may inhibit rates of glucose absorption, in a similar way to phlorizin (a dihydrochalcone in apple that inhibits SGLT1-mediated glucose uptake) [26], [27].

The strong inhibition of plasma GIP concentrations throughout the postprandial period suggests that secretory regulation of this incretin is highly sensitive to glucose absorption rates. SGLT1 has been shown to be coexpressed with GLP-1 and GIP in individual enteroendocrine cells of murine jejunal crypts, and studies in *Sglt1*^*−/−*^ mice have shown the essential role of SGLT1 as a glucose sensor for GIP and GLP-1 secretion [28], although GLUT2 may also be an important regulator of incretin response [29]. Our human data showing inhibition of carbohydrate-induced incretin secretion following consumption of blackcurrant polyphenols mirror previous reports of phlorizin sensitivity of glucose-induced SGLT1-mediated incretin release [30].

The suppression of plasma GLP-1 concentrations by the top dose of blackcurrant extract paradoxically coincided with the time point where plasma insulin concentrations were increased relative to placebo, at 90 min postdrink. Polyphenol-induced delayed digestive processing of the starch/sucrose test meal is likely to have resulted in partially digested dextrins and disaccharides possibly shifting further down the small intestine. This may have increased the proportion of glucose that was absorbed later (75–90 min) relative to control accounting for the crossover in glucose and insulin profiles, in agreement with glycaemic/insulinaemic profiles observed previously [15], [16], [17]. GLP-1 is released from L cells in the distal intestine within minutes of nutrient ingestion *via* neuroendocrine signalling, but inhibition by blackcurrant extract occurred around the time that the meal contents would be reaching the part of the jejunum where the secretory cells are located suggesting a direct inhibition of glucose transport in this part of the gut by the blackcurrant polyphenols.

No acute differences in blood pressure, arterial stiffness or pulse wave reflection index as measured by DVP were observed between drinks, consistent with previous vascular observations following single meals [31], [32]. This does not exclude the possibility that there might be an effect of blackcurrant polyphenols on vascular function. Anthocyanins rapidly appear in the circulation 1–2 h after consumption at very low concentrations (1–150 nmol/l for intakes of 3–1200 mg) [33], [34] and are equally rapidly cleared, so are unlikely to have a significant impact on vascular function early in the postprandial period [35], although blackcurrant phenolic acids that peak around 2 h post ingestion might be associated with some improvement in endothelial function [31]. However, colonic metabolites, conjugated phenolic acids, would be expected to appear in the circulation 4–12 h post ingestion and may also be bioactive in this respect [33]. Acute episodes of high blood glucose cause endothelial dysfunction in animal models and healthy volunteers in clinical trials, with hyperglycemia-induced oxidative stress as the most likely mediator [19], [36]. However, the different profiles of postprandial glycemia observed here were not associated with any significant differences in a sensitive marker of lipid peroxidation, 8-isoprostane F_2α_.

The strengths of this study include the rigorous adherence to double blinding until final statistical analysis was complete, randomization and allocation concealment being conducted at a remote site by an independent party and the control drink was fully matched to the intervention drinks for taste, appearance and nutrient composition. The study was carried out in a broad cross-section of healthy men and postmenopausal women, although premenopausal women were excluded to avoid confounding effects of reproductive hormones [37]. Limitations of the study include the fact that the difficulty in finding a bitter/astringent taste additive that is not a potentially bioactive compound meant that tannins were added to placebo and lower doses of blackcurrant extract drinks for taste blinding. Although we observed significant inhibition of glycemia, insulinemia and incretin secretion, the true size of the effect may have been much larger if the requirement for blinding had not necessitated addition of tannins. Serum concentrations of anthocyanins and their metabolites were not measured in this study since the primary aim was to investigate effects on postprandial glycaemia, mainly determined by polyphenol interactions with digestive enzymes and intestinal glucose transporters within the lumen of the gastrointestinal tract. However, interpretation of some of the secondary outcome variables (vascular function and blood pressure) would have been aided by data on circulating blackcurrant polyphenol derivatives.

In conclusion, anthocyanin-rich blackcurrant extract, providing a similar quantity of polyphenols to that found in five heaped tablespoons of blackcurrants, delayed the appearance of glucose in the blood and inhibited the secretion of insulin and incretins. Although blackcurrants are likely to be consumed only sporadically in the general population, other berries contain different profiles of anthocyanins. Regular consumption of any combination of these as main meals or with snack foods may help lower postprandial glycemia which may reduce the vascular burden of glucose-induced oxidative stress and endothelial dysfunction [19], [20] involved in the progression of atherosclerosis. This evidence supports current dietary advice that a predominantly plant-based diet may help prevent CVD and T2D and suggests one of many routes by which cardio-metabolic protection can occur.

The following is the supplementary data related to this article.

Supplementary Fig. 1 Postprandial plasma TAG and NEFA concentrations. Mean (±S.E.M.) plasma TAG (A) and NEFA (B) concentrations following ingestion of four low sugar fruit drinks 2 min before consuming a mixed carbohydrate meal, in randomized order: H-BE: high blackcurrant extract (600-mg anthocyanins); M-BE: medium blackcurrant extract (300-mg anthocyanins); L-BE: low blackcurrant extract (150-mg anthocyanins); CON: placebo (0 mg anthocyanins). *n*=22. All data were natural log transformed before mixed model analysis. For TAG, there was a treatment effect (*P*<.0000001) on raw values but not for changes from baseline, and no treatment × time interactions was observed. There were no statistically significant treatment differences in NEFA concentrations. A: ^a^*P*<0.005 for the treatment difference at baseline. Supplementary Fig. 1

## Figures and Tables

**Table 1:** Energy, nutrient and polyphenol composition of the test drinks

Per 200 ml	H-BE	M-BE	L-BE	CON
Energy and nutrients				
Energy, *kJ*	92	90	90	95
Carbohydrate, *g*	4.6	4.5	4.5	4.8
Glucose, *g*	0.2	0.2	0.2	0.2
Fructose, *g*	0.2	0.2	0.2	0.2
Sucrose/Lactose/Maltose, *g*	<0.1	<0.1	<0.1	<0.1
Starch, *g*	4.0	4.0	5.0	4.0
Fat, *g*	0.4	0.4	0.4	0.4
Protein, *g*	<0.1	<0.1	<0.1	<0.1
Total dietary fibre (AOAC), *g*	<0.5	<0.5	<0.5	<0.5
Vitamin C, mg	<0.5	<0.5	<0.5	<0.5
Vitamin E, mg	<0.2	<0.2	<0.2	<0.2
Sodium, mg	<3	<3	<3	<3
Potassium, mg	15.7	7.8	3.9	<3
Magnesium, mg	2.3	1.1	0.6	<0.4
Calcium, mg	6.5	3.3	<3	<3
Phosphorus, mg	<2	<2	<2	<2
Zinc, mg	<0.1	<0.1	<0.1	<0.3
Polyphenols				
Total phenolics, mg^1^, ^2^	1596	810	460	207
Total anthocyanins, mg^1^	599	322	131	46
Delphinidin-3-rutinoside, mg^3^	260	140	57	20
Cyanidin-3-rutinoside, mg^3^	209	113	46	16
Delphinidin-3-glucoside, mg^3^	76	41	17	6
Cyanidin-3-glucoside, mg^3^	33	18	7	3

H-BE: high blackcurrant extract; M-BE: medium blackcurrant extract; L-BE: low blackcurrant extract; CON: no blackcurrant extract.

1 Total phenolic and anthocyanin content estimated from direct analysis of drinks by Folin-Ciocalteau method and HPLC respectively.

2 Tannins added as an ingredient (H-BE 0 mg, M-BE 60 mg, L-BE 90 mg, CON 150 mg per 200-ml drink) to render drinks equivalent in bitter/astringent taste for blinding purposes.

3 Estimated from HPLC analysis of raw extract, not direct analysis of the drinks.

**Table 2:** Characteristics of healthy subjects who completed the study (n=22)^a^

Variable	
Age, year	45.4 (13.7)
Sex, male to female ratio	13:9
Body mass index, kg/m^2^	25.5 (3.8)
Systolic blood pressure, mmHg	122.7 (14.1)
Diastolic blood pressure, mmHg	77.2 (10.8)
Waist circumference, cm	
Males	88.3 (9.6)
Females	89.2 (13.9)
Body fat, *%*	
Males	18.1 (6.2)
Females	35.1 (8.2)
Fasting plasma glucose, mmol/l	5.4 (0.5)
Fasting plasma triacylglycerol, mmol/l	1.2 (0.5)
Fasting plasma total cholesterol, mmol/l	4.9 (1.0)
Fasting plasma LDL cholesterol, mmol/l	2.8 (0.8)
Fasting plasma HDL cholesterol, mmol/l	1.5 (0.4)
Males	1.3 (0.4)
Females	1.7 (0.3)

a Values are means (S.D.).

**Table 3:** Effects of blackcurrant extract and placebo test drinks on digital volume pulse (DVP), systolic and diastolic blood pressure (SBP and DBP) and plasma 8-isoprostane F_2α_ in healthy men and postmenopausal women^1^

		CON	L-BE	M-BE	H-BE
DVP-RI, %	Baseline	74.4 (69.5, 79.2)	70.4 (64.8, 76.1)	72.9 (67.1, 78.6)	72.8 (68.0, 77.5)
	Δ120 min	-0.57 (−5.35, 4.21)	3.80 (−0.93, 8.54)	0.15 (−4.26, 4.56)	1.38 (−3.28, 6.04)
DVP-SI, m/s	Baseline	8.57 (7.75, 9.40)	8.50 (7.63, 9.37)	8.44 (7.64, 9.25)	8.71 (7.62, 9.80)
	Δ 120 min	-0.75 (−1.44, −0.05)	-0.23 (−1.05, 0.59)	-0.57 (−1.21, 0.07)	-0.55 (−1.54, 0.45)
SBP, mmHg	Baseline	120.8 (116.1, 125.2)	120.9 (115.6, 126.0)	120.7 (116.0, 125.5)	118.3 (113.7, 122.9)
	Δ 120 min	-4.5 (−8.8, −0.1)	-1.0 (−6.8, 4.9)	-5.2 (−8.9, −1.5)	-1.7 (−6.7, 3.3)
DBP, mmHg	Baseline	74.6 (71.2, 78.1)	75.3 (71.7, 78.9)	74.5 (71.3, 77.8)	73.6 (70.8, 76.4)
	Δ 120 min	-0.6 (−4.3, 3.2)	1.7 (−2.0, 5.4)	-1.1 (−3.6, 1.3)	0.5 (−2.5, 3.5)
Plasma 8-isoprostane F_2α_, pmol/L^2^	Baseline	86.4 (68.8, 108.4)^3^	123.7 (96.6, 158.3)^3^	100.5 (80.8, 125.1)	98.4 (76.6, 126.2)
	Δ 120 min	1.13 (1.01, 1.27)	0.94 (0.81, 1.09)	1.02 (0.91, 1.14)	0.97 (0.85, 1.11)

H-BE: high blackcurrant extract (600-mg anthocyanins); M-BE: medium blackcurrant extract (300-mg anthocyanins); L-BE: low blackcurrant extract (150-mg anthocyanins); CON: placebo (0 mg anthocyanins). No significant differences were observed between drinks.

1 Values are mean baseline values and changes from baseline measured 120 min after the test drink and mixed carbohydrate meal (95% CI), *n*=22.

2 Values are geometric mean baseline values and log ratios of changes from baseline measured 120 min after the test drink and mixed carbohydrate meal (95% CI), *n*=22.

3 *n*=20 due to sample loss.
