# Supplementary material for: Drinks containing anthocyanin-rich blackcurrant extract decrease postprandial blood glucose, insulin and incretin concentrations
Source: J Nutr Biochem. 2016 Dec;38:154–61. doi: 10.1016/j.jnutbio.2016.09.002 (PMC5170886; doi:10.1016/j.jnutbio.2016.09.002)
Supplement: Supplementary Fig. 1 — Postprandial plasma TAG and NEFA concentrations. Mean (±S.E.M.) plasma TAG (A) and NEFA (B) concentrations following ingestion of four low sugar fruit drinks 2 min before consuming a mixed carbohydrate meal, in randomized order: H-BE: high blackcurrant extract (600-mg anthocyanins); M-BE: medium blackcurrant extract (300-mg anthocyanins); L-BE: low blackcurrant extract (150-mg anthocyanins); CON: placebo (0 mg anthocyanins). n=22. All data were natural log transformed before mixed model analysis. For TAG, there was a treatment effect (P<.0000001) on raw values but not for changes from baseline, and no treatment × time interactions was observed. There were no statistically significant treatment differences in NEFA concentrations. A: aP<0.005 for the treatment difference at baseline. [file mmc1.pdf]

A

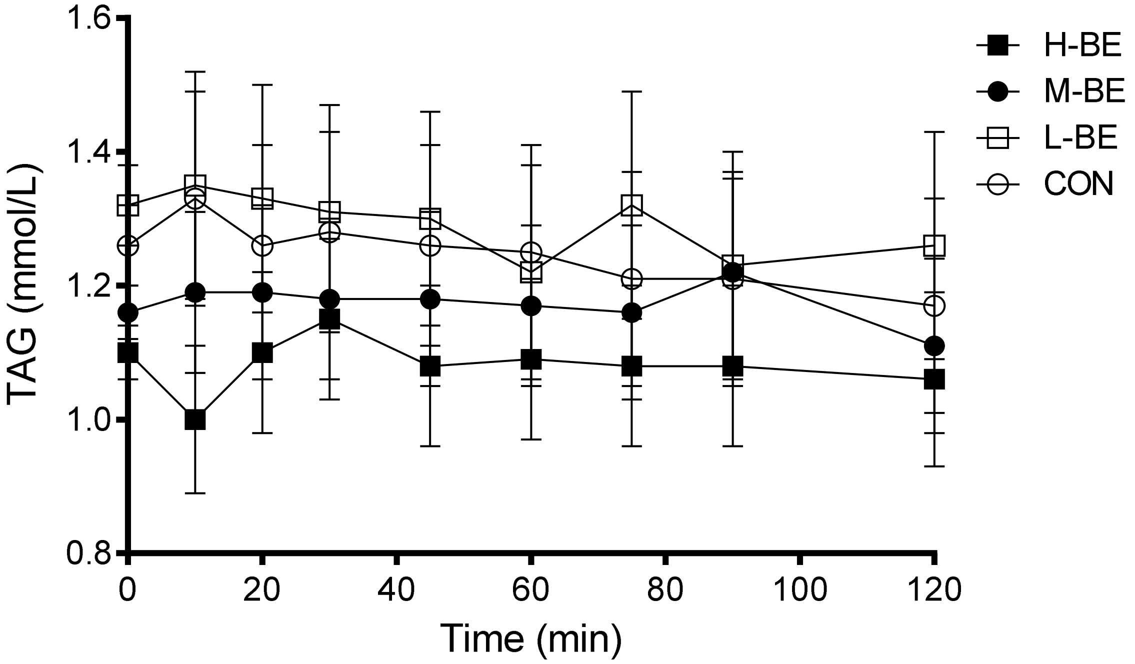

B

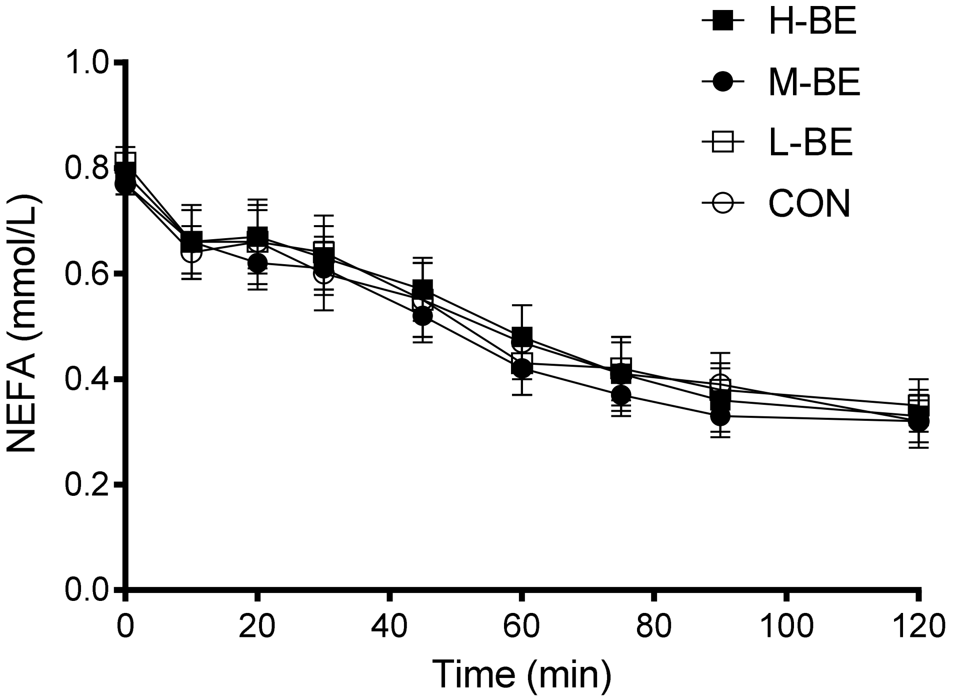

Supplementary Figure 1: Postprandial plasma TAG and NEFA concentrations

Mean (±SEM) plasma TAG (**A**) and NEFA (**B**) concentrations following ingestion of 4 low sugar fruit drinks 2 minutes before consuming a mixed carbohydrate meal, in randomized order: H-BE: high blackcurrant extract (600 mg anthocyanins); M-BE: medium blackcurrant extract (300 mg anthocyanins); L-BE: low blackcurrant extract (150 mg anthocyanins); CON: placebo (0 mg anthocyanins). n = 22. All data were natural log transformed before mixed model analysis. For TAG, there was a treatment effect ($P < 0.0000001$) on raw values but not for changes from baseline and no treatment x time interactions was observed. There were no statistically significant treatment differences in NEFA concentrations. **A**: $^{a}P < 0.005$ for the treatment difference at baseline.
